# Radiomics analysis enhances the diagnostic performance of CMR stress perfusion: a proof-of-concept study using the Dan-NICAD dataset

**DOI:** 10.3389/fcvm.2023.1141026

**Published:** 2023-09-15

**Authors:** Zahra Raisi-Estabragh, Carlos Martin-Isla, Louise Nissen, Liliana Szabo, Victor M. Campello, Sergio Escalera, Simon Winther, Morten Bøttcher, Karim Lekadir, Steffen E. Petersen

**Affiliations:** ^1^ William Harvey Research Institute, NIHR Barts Biomedical Research Centre, Queen Mary University of London, London, United Kingdom; ^2^ Barts Heart Centre, St Bartholomew’s Hospital, Barts Health NHS Trust, London, United Kingdom; ^3^ Dept. de Matematiques I Informatica, University of Barcelona, Barcelona, Spain; ^4^ Department of Cardiology, Regionshospital Gødstrup, Herning, Denmark; ^5^ Heart and Vascular Center, Semmelweis University, Budapest, Hungary; ^6^ Departament de Matemàtiques & Informàtica, Universitat de Barcelona, Barcelona, Spain; ^7^ Computer Vision Center, Univeritat Autònoma de Barcelona, Barcelona, Spain; ^8^ Health Data Research UK, London, United Kingdom; ^9^ Alan Turing Institute, London, United Kingdom

**Keywords:** radiomics, Dan-NICAD, CMR (cardiovascular magnetic resonance), stress perfusion cardiac MRI, machine learning (ML)

## Abstract

**Objectives:**

To assess the feasibility of extracting radiomics signal intensity based features from the myocardium using cardiovascular magnetic resonance (CMR) imaging stress perfusion sequences. Furthermore, to compare the diagnostic performance of radiomics models against standard-of-care qualitative visual assessment of stress perfusion images, with the ground truth stenosis label being defined by invasive Fractional Flow Reserve (FFR) and quantitative coronary angiography.

**Methods:**

We used the Dan-NICAD 1 dataset, a multi-centre study with coronary computed tomography angiography, 1,5 T CMR stress perfusion, and invasive FFR available for a subset of 148 patients with suspected coronary artery disease. Image segmentation was performed by two independent readers. We used the Pyradiomics platform to extract radiomics first-order (*n* = 14) and texture (*n* = 75) features from the LV myocardium (basal, mid, apical) in rest and stress perfusion images.

**Results:**

Overall, 92 patients (mean age 62 years, 56 men) were included in the study, 39 with positive FFR. We double-cross validated the model and, in each inner fold, we trained and validated a per territory model. The conventional analysis results reported sensitivity of 41% and specificity of 84%. Our final radiomics model demonstrated an improvement on these results with an average sensitivity of 53% and specificity of 86%.

**Conclusion:**

In this proof-of-concept study from the Dan-NICAD dataset, we demonstrate the feasibility of radiomics analysis applied to CMR perfusion images with a suggestion of superior diagnostic performance of radiomics models over conventional visual analysis of perfusion images in picking up perfusion defects defined by invasive coronary angiography.

## Introduction

Cardiovascular risk stratification of subjects with suspected cardiovascular disease is of crucial public health importance. The management of this cohort is mostly informed by the assessment of traditional vascular risk factors. However, recently stress perfusion cardiovascular magnetic resonance (CMR) has emerged as an important non-invasive test for assessment of myocardial ischaemia (1). Typically, left ventricular (LV) slices are imaged to capture first-pass perfusion of gadolinium contrast during adenosine stress. Image interpretation relies on assessment of visually detectable difference in signal intensities (SIs) between areas of normal and abnormal perfusion. Invasive functional flow reserve (FFR) is the reference standard for assessment of flow-limiting coronary artery disease (CAD) (2). The diagnostic accuracy of stress myocardial stress perfusion CMR against FFR as the reference standard has, at times, been suboptimal (3).

CMR radiomics is an emerging image analysis method allowing derivation of a multitude of quantitative features from existing images (4). Radiomics SI-based features (first-order, texture) describe the global distribution (e.g., mean, variance, entropy, energy) and inter-pixel relationships (e.g., autocorrelation, cluster prominence, contrast, run length emphasis) of intensity levels. Extracted from the LV myocardium they quantitatively characterise the distribution and pattern of myocardial SIs, to a greater granularity than can be appreciated visually by human experts. Clinical models developed using CMR radiomics features have shown promising results for disease discrimination (4). Application of radiomics analysis to CMR perfusion images may provide a novel quantitative and automated method for image analysis, which may improve diagnostic accuracy. The feasibility of this approach has not been previously reported.

In the present study, we first assess the feasibility of extracting radiomics SI-based features from the myocardium in CMR stress perfusion sequences and, second, we compare the diagnostic performance of radiomics models against conventional image analysis.

## Methods

### Study participants and material

We used the Dan-NICAD 1 dataset (3), a multi-centre study with coronary computed tomography angiography, 1,5 tesla CMR stress perfusion, and invasive FFR available for a subset of 148 patients with suspected CAD. Stress induction was conducted using intravenous injection of either 0.4 mg (5 ml) of Regadenosone (Lexiscan, Astellas Pharma, USA) or intravenous Adenosine infusion of 140 g/kg/min over 4 min. A stepwise increase in adenosine dose was administered in case of inadequate response and splenic switch-off was registered in all patients stressed with adenosine (3). Conventional CMR analysis result was taken as the disease classification assigned to each case in expert analysis of the original study. The reference standard was based on FFR and invasive quantitative coronary angiography (QCA). A positive case was indicated by either FFR < 0.8 or coronary stenosis >90% on QCA, with results reported per-patient and per-vessel (which we converted to per-territory) to match the Dan-NICAD study protocol (5).

### CMR protocol and post-processing analysis

Image segmentation was performed blind to all patient details by two independent readers (ZRE, SEP) using CVI42 software (Version 5.1.1, Circle Cardiovascular Imaging Inc., Calgary, Canada). The LV myocardium from short axis perfusion images was segmented using a semi-automated method. All images were inspected with manual adjustment as needed, care was taken to ensure close apposition of contours to the epi- and endocardium. In order to ensure the accuracy and reliability of the radiomics analysis, we excluded cases without matching rest and stress images, any cases with motion or breathing artefact, very high basal slices, lack of temporality, or with missing per-territory diagnosis result.

### Radiomics feature extraction and selection

**Figure 1:**
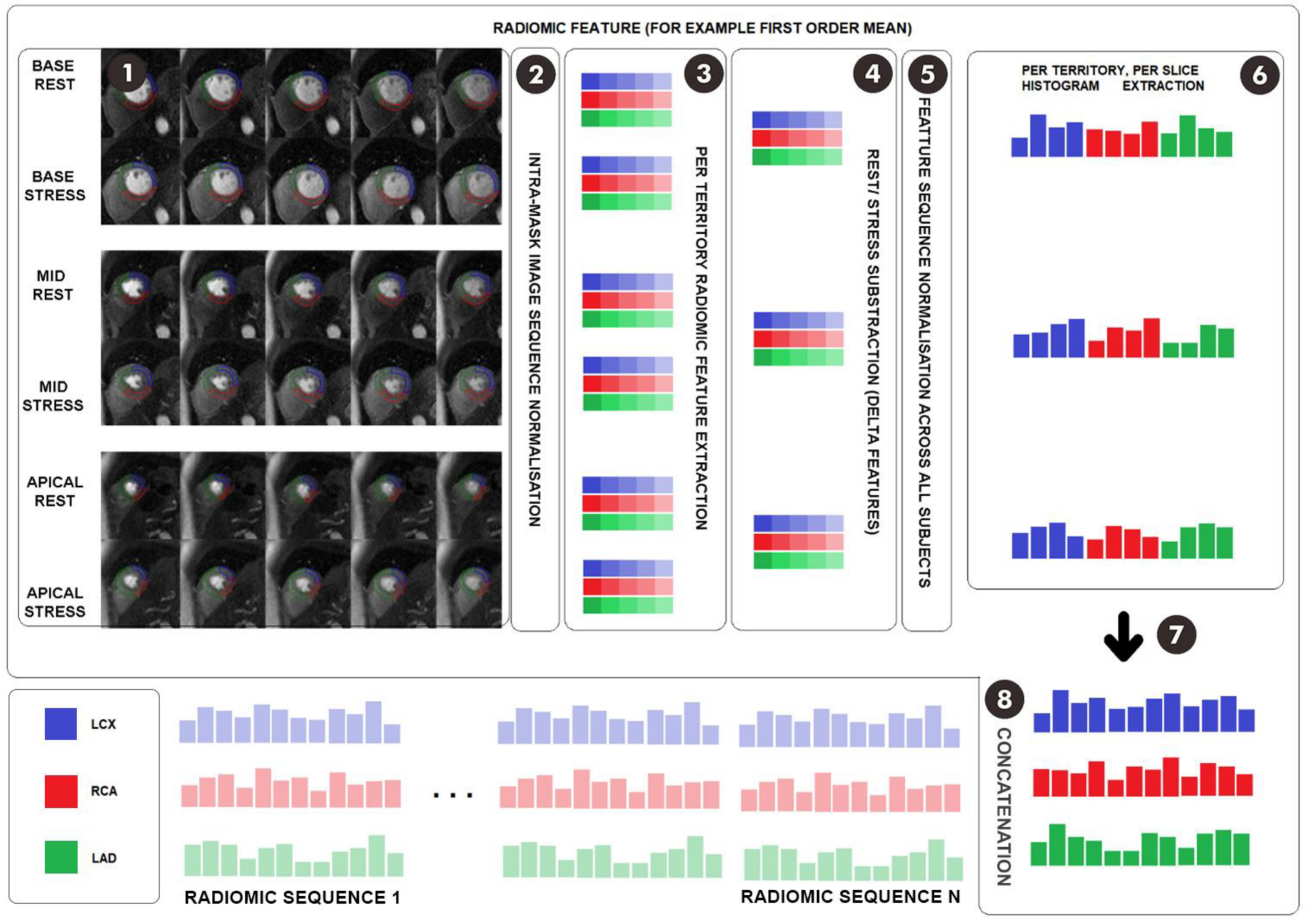
Feature extraction scheme. Footnote: (1) Temporal stress and rest acquisitions for basal, mid, and apical planes are delineated into coronary territories. (2) Temporal image sequences are min-max normalized along all timesteps to preserve the contrast effect. (3) For each of the six territory sequences, the desired feature is extracted at each timestep, obtaining six feature sequences. (4) Stress and rest feature sequences are subtracted for each region and plane, thus reducing dimensionality. (5) Temporal sequences are min-max normalized to avoid negative values, adding an extra standardization layer. (6) Temporal sequences are converted to histograms to avoid inter-subject contrast effect misalignment. (7) Histograms are reordered to regroup LCX, LAD and RCA histogram feature sequences, thus allowing per-territory analysis. (8) The process is repeated for each radiomic feature and the histogram sequences are concatenated. LCX, left circumflex artery; LAD, left anterior descending; RCA, right coronary artery.

We used the open source Pyradiomics (6) platform to extract radiomics first-order (*n* = 14) and texture (*n* = 75) features from the LV myocardium (basal, mid, apical) in rest and stress perfusion images. Features were extracted independently from each slice to avoid 3D inconsistencies. To reduce variation in SIs due to the acquisition process, we applied image normalisation per sequence and per slice. To reduce noise, we used the arterial input function curves to crop the time interval included in the modelling; this was set to include the first SI peak and the cycles immediately either side. We subtracted stress and rest radiomics features from each corresponding LV slice and territory, obtaining a total of nine sequences per subject. We transformed time sequences (delta sequences) to histograms to avoid inter-subject misalignment and contrast effect skewness. Each case was split into three territories with individual assessment. We assessed per-territory with each territory having three sequences, one for the corresponding region of each slice to that territory. The three sequences for each territory were concatenated. We first removed highly correlated values, resulting in 19 first-order features and 16 Gray Level Size Zone Matrix (GLSZM) features, totaling 35 unique features. Each temporal sequence was interpolated to 10 timesteps, leading to a total of 2,100 features. After performing delta subtraction, we were left with half the number of features, which was 1,050. We further reduced the number of histogram bins to 8 bins per radiomics sequence. Therefore, in the final experiment, we had a total of 840 features (35 features x 8 histogram bins x 3 slices). Due to the high dimensionality resulting from the sequences, we performed sequential feature selection of up to 50 features in the inner fold of the nested cross-validation. This allowed us to select the most relevant features associated with the ground truth, while avoiding overfitting and maintaining a manageable feature set for model training and evaluation. Radiomics feature extraction is shown on (Figure 1).

### Modelling

We developed the radiomics model using a balanced random forest algorithm, combined with nested cross-validation, to address the potential issue of class imbalance and ensure a robust model evaluation. The process involved the following steps: (1) In each inner fold, we performed sequential feature selection using a balanced random forest algorithm with 10-fold cross-validation. This step allowed us to select the most informative features for the model. (2) After selecting the best features, we conducted a grid search to optimize the hyperparameters of the balanced random forest model, again using 10-fold cross-validation. This ensured that the model would perform optimally with the selected features. (3) To avoid data leakage and overfitting, we used Stratified Group K-Fold cross-validation for both feature selection and hyperparameter optimization. Territories of the same subject were grouped together, either in training or validation sets, to maintain the independence of the samples. (4) Finally, the fine-tuned model with the best parameters was applied to the territories of the subjects in the outer folds, with the territories also grouped to avoid data leakage from the inner folds.

## Results

**Figure 2:**
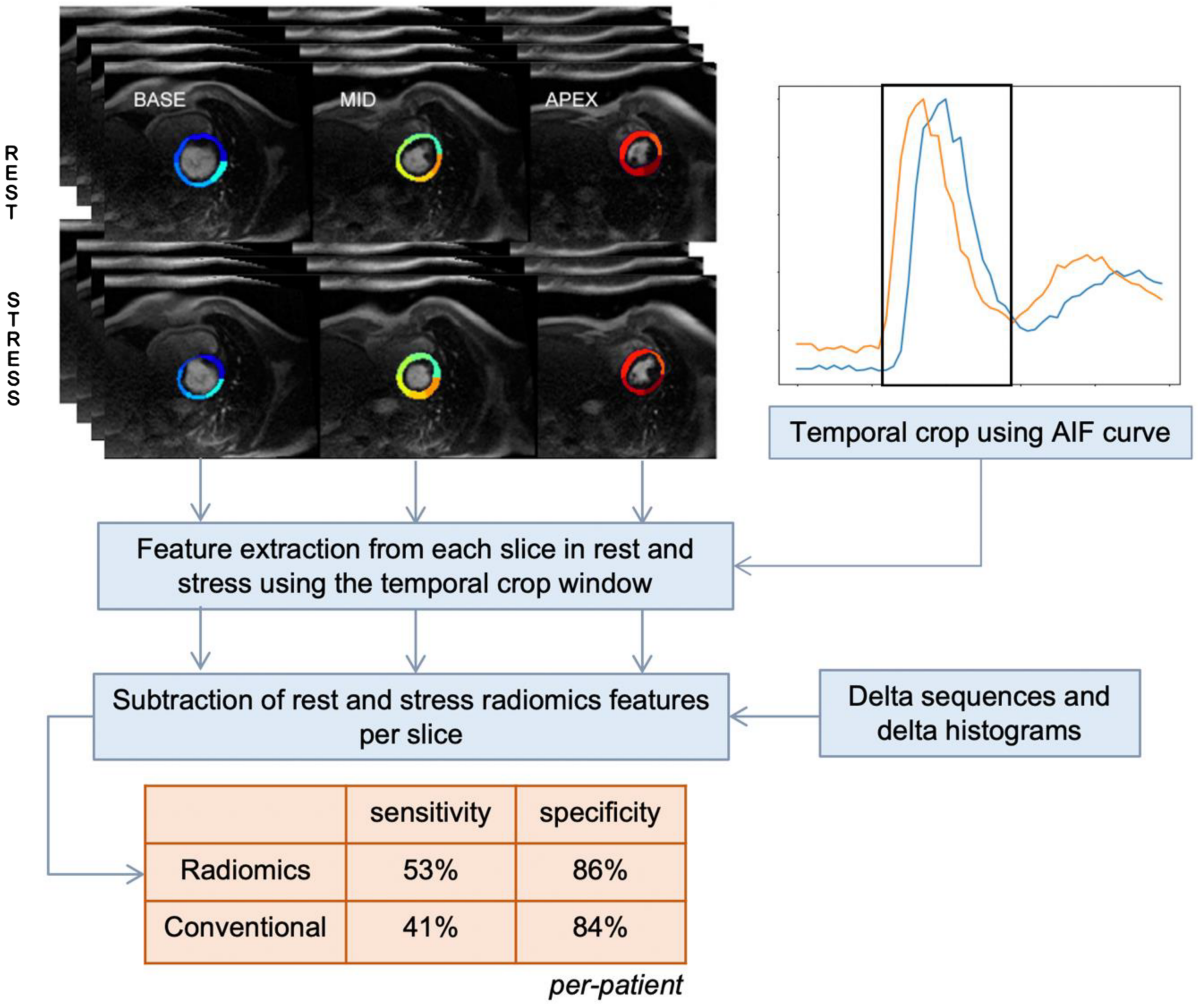
Central illustration. Summary of study workflow and key results. AIF, arterial input function.

We included 92 patients in the study, with average age of 62 years, including 56 (61%) men, and 39 (42%) with positive FFR (147 positive and 129 negative territories).We double-cross validated the model and, in each inner fold, we trained and validated a per territory model (with FFR/QCA result as the reference standard). The threshold probability that matched the global specialist's specificity was obtained from each validation set. In the outer test folds diagnostic probabilities for each territory were obtained and a case with positive result in any territory was assigned “positive”. That is, in the inner fold we chose to adopt a per-territory analysis to increase the sample size and reduce imbalance of positive/negative cases. The assessment in the outer fold (the test fold) outputted a per-patient result if any of the territories for that case were assigned positive. The conventional analysis results reported sensitivity of 41% and specificity of 84% (Figure 2). Granular results from our radiomics models are presented in Table 1. Our final radiomics model demonstrated a significant improvement on these results with an average sensitivity of 53% and specificity of 86% (Table 1). We compared clinical binary outcomes and rounded prediction probabilities obtained by our method using McNemar test, which showed a significant difference between the two methods (test statistics 11.0, *p*-value: 0.0409). To aide interpretation we conducted a feature ranking analysis to summarize the most frequently selected feature types across the folds (Supplementary Figure 1). In our analysis, the three features most significantly associated with the detection of perfusion defects were identified as follows: First Order Median, a measure derived from the intensity histogram of the image; GLSZM High Gray Level Zone Emphasis, which characterizes the distribution of zones with high pixel intensity within the image; and GLSZM Large Area Emphasis, a metric that highlights larger areas of the image exhibiting homogeneous gray level intensity.

**Table 1:** Granular results from CMR stress perfusion radiomics analysis.

	Sensitivity	Specificity
Model 1 - Per patient	35%	85%
Model 2 - Per territory (no delta features)	40%	86%
Model 3 - Per territory (delta features)	44%	86%
Model 4 - Per territory (delta to histogram)	53%	86%

Here we detail the results from our radiomics models. Model 1) per patient model, which corresponds to the features extracted for each frame and slice without territory division. Model 2) per territory model, which corresponds to the features extracted for each frame, a slice and territory. Model 3) delta features model, which correspond to the subtraction of stress and rest normalized radiomic sequences. This is min-max normalisation of the same sequence along all subjects. Final model, which contains Model 3) and plus the transformation of the normalized radiomic sequences to histograms of 8 bins.

## Discussion

In this proof-of-concept study of 92 participants from the Dan-NICAD 1 dataset, we demonstrate the feasibility of radiomics analysis applied to CMR perfusion images with suggestion of a superior diagnostic performance of radiomics models over conventional analysis.

To date the clinical assessment of CMR stress perfusion has utilized visual judgment of ischaemic segments which lead to several key limitations including underestimation of ischemic burden (7) or inadequate discrimination of cardiac microvascular disease. This has resulted in efforts to standardized and objectively quantify myocardial perfusion. Of note, most novel quantitative perfusion mapping tools requires specialized sequences and protocol (7). Our pipeline on the other hand enabled the extraction of pixel level tissue information from conventional perfusion CMR images, permitting the assessment of a richer and objective perfusion data over visual assessment without requiring additional sequences or modification of protocol.

In our study, we observed a slightly higher sensitivity (53%) in identifying positive CAD cases through radiomics analysis compared to conventional stress perfusion imaging (41%). This improvement, although marginal, offers promising implications for future non-invasive ischemia detection. However, the sensitivity of our radiomics approach, while improved, remains relatively modest. This limitation echo prior findings from the Dan-NICAD cohort. As highlighted by Nissen et al. (3), key discrepancies exist between the Dan-NICAD study and previous reports like CE-MARC (8) in terms of stress CMR's diagnostic power. These disparities stem primarily from differences in patient population, disease definition, and selection criteria; most importantly in the Dan-NICAD trial patients were selected using coronary computed tomography angiography. These aspects necessitate a cautious interpretation of our study's sensitivity, warranting further refinements in the radiomics approach to optimize its performance within diverse clinical contexts. At the same time, we reached similar specificity as reported in the literature (9). The reported marginal improvement over the conventional stress perfusion assessment promises to improve non-invasive ischaemia detection based on conventional sequences in the future, even in imaging centers where novel CMR machines or sequence packages are less accessible than in leading institutions.

We employed feature importance analysis, a common machine learning interpretability technique, to quantify the contribution of each radiomic feature towards differentiating cases with perfusion defects from those without. Our analysis pinpointed the First Order Median, GLSZM High Gray Level Zone Emphasis, and GLSZM Large Area Emphasis as the most consequential features for the detection of perfusion defects. The importance of the First Order Median suggests that variations in tissue properties, as reflected in median pixel intensity, play a pivotal role in distinguishing between positive and negative territories. The significance of GLSZM High Gray Level Zone Emphasis underscores the potential influence of both the distribution of high-intensity zones and the size of homogeneous regions on the predictability of ischemic territories (10). These observations could steer future research towards a deeper exploration of the pathological mechanisms underlying these associations, which could enhance the clinical utility of our radiomics model.

Our study has the following limitations: first this proof-of-concept study was conducted in a relatively small cohort and would benefit from reproducing in a larger sample. Second, the sensitivity of our radiomics method currently stands at 53%, which might limit its immediate utility in routine clinical practice due to the potential risk of false negatives. This finding underscores the potential inherent limitations of the imaging technique and the need for further refinement and optimization of our models before widespread clinical implementation can be considered. Third, our study would benefit from external validation what could provide broader insights into the real-world performance of our model.

## Data Availability

The data analyzed in this study is subject to the following licenses/restrictions: Data accessibility statement available at ClinicalTrials.gov (Identifier: NCT02264717). Requests to access these datasets should be directed to Louise Nissen lounisse@rm.dk.

## References

[1] Gulati M Levy PD Mukherjee D Amsterdam E Bhatt DL Birtcher KK 2021 AHA/ACC/ASE/CHEST/SAEM/SCCT/SCMR guideline for the evaluation and diagnosis of chest pain: a report of the American college of cardiology/American heart association joint committee on clinical practice guidelines. Circulation. (2021) 144:e368–454. 10.1161/CIR.0000000000001029 34709879

[2] Berry C Corcoran D Hennigan B Watkins S Layland J Oldroyd KG. Fractional flow reserve-guided management in stable coronary disease and acute myocardial infarction: recent developments. Eur Heart J. (2015) 36:3155–64. 10.1093/eurheartj/ehv206 26038588 PMC4816759

[3] Nissen L Winther S Westra J Ejlersen JA Isaksen C Rossi A Diagnosing coronary artery disease after a positive coronary computed tomography angiography: the Dan-NICAD open label, parallel, head to head, randomized controlled diagnostic accuracy trial of cardiovascular magnetic resonance and myocardial perfusion s. Eur Hear J Cardiovasc Imaging. (2018) 19:369–77. 10.1093/ehjci/jex342 29447342

[4] Raisi-Estabragh Z Izquierdo C Campello VM Martin-Isla C Jaggi A Harvey NC Cardiac magnetic resonance radiomics: basic principles and clinical perspectives. Eur Heart J Cardiovasc Imaging. (2020) 21:349–56. 10.1093/ehjci/jeaa028 32142107 PMC7082724

[5] Nissen L Winther S Isaksen C Ejlersen JA Brix L Urbonaviciene G Danish study of non-invasive testing in coronary artery disease (Dan-NICAD): study protocol for a randomised controlled trial. Trials. (2016) 17:1–11. 10.1186/s13063-016-1388-z 27225018 PMC4880871

[6] Van Griethuysen JJM Fedorov A Parmar C Hosny A Aucoin N Narayan V Computational radiomics system to decode the radiographic phenotype. Cancer Res. (2017) 77:e104–7. 10.1158/0008-5472.CAN-17-0339 29092951 PMC5672828

[7] Kotecha T Chacko L Chehab O O’Reilly N Martinez-Naharro A Lazari J Assessment of multivessel coronary artery disease using cardiovascular magnetic resonance pixelwise quantitative perfusion mapping. JACC Cardiovasc Imaging. (2020) 13:2546–57. 10.1016/j.jcmg.2020.06.041 33011115

[8] Greenwood JP Maredia N Younger JF Brown JM Nixon J Everett CC Cardiovascular magnetic resonance and single-photon emission computed tomography for diagnosis of coronary heart disease (CE-MARC): a prospective trial. Lancet. (2012) 379:453–60. 10.1016/S0140-6736(11)61335-4 22196944 PMC3273722

[9] Patel AR Salerno M Kwong RY Singh A Heydari B Kramer CM. Stress cardiac magnetic resonance myocardial perfusion imaging: JACC review topic of the week. J Am Coll Cardiol. (2021) 78:1655–68. 10.1016/j.jacc.2021.08.022 34649703 PMC8530410

[10] Scapicchio C Gabelloni M Barucci A Cioni D Saba L Neri E. A deep look into radiomics. Radiol Medica. (2021) 126:1296–311. 10.1007/s11547-021-01389-x PMC8520512 34213702

