# Supplementary figures and images for: Radiomics analysis enhances the diagnostic performance of CMR stress perfusion: a proof-of-concept study using the Dan-NICAD dataset
Source: Front Cardiovasc Med. 2023 Sep 15;10:1141026. doi: 10.3389/fcvm.2023.1141026 (PMC10541220; doi:10.3389/fcvm.2023.1141026)

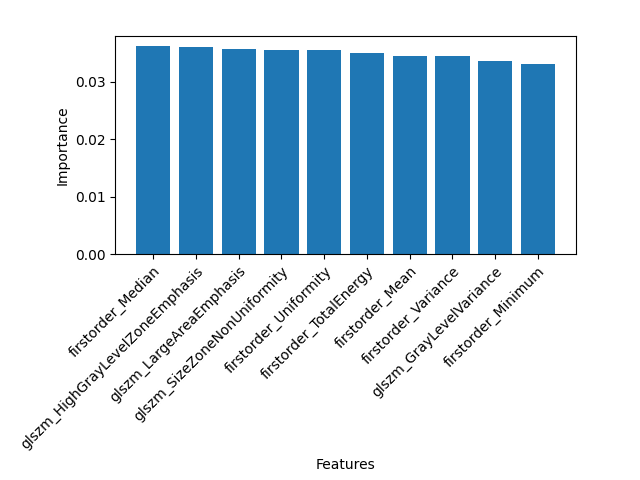

Supplement: Supplementary file 1 [file Image1.png]
